# An Association Between ICP-Derived Data and Outcome in TBI Patients: The Role of Sample Size

**DOI:** 10.1007/s12028-016-0319-x

**Published:** 2016-11-07

**Authors:** Brenno Cabella, Joseph Donnelly, Danilo Cardim, Xiuyun Liu, Manuel Cabeleira, Peter Smielewski, Christina Haubrich, Peter J. A. Hutchinson, Dong-Joo Kim, Marek Czosnyka

**Affiliations:** 1 Division of Neurosurgery, Department of Clinical Neurosciences, University of Cambridge, Cambridge, UK; 2 Department of Brain and Cognitive Engineering, Korea University, Seoul, South Korea

**Keywords:** Traumatic brain injury, Outcome prediction, Statistical inference, Intracranial pressure, Autoregulation

## Abstract

**Background:**

Many demographic and physiological variables have been associated with TBI outcomes. However, with small sample sizes, making spurious inferences is possible. This paper explores the effect of sample sizes on statistical relationships between patient variables (both physiological and demographic) and outcome.

**Methods:**

Data from head-injured patients with monitored arterial blood pressure, intracranial pressure (ICP) and outcome assessed at 6 months were included in this retrospective analysis. A univariate logistic regression analysis was performed to obtain the odds ratio for unfavorable outcome. Three different dichotomizations between favorable and unfavorable outcomes were considered. A bootstrap method was implemented to estimate the minimum sample sizes needed to obtain reliable association between physiological and demographic variables with outcome.

**Results:**

In a univariate analysis with dichotomized outcome, samples sizes should be generally larger than 100 for reproducible results. Pressure reactivity index, ICP, and ICP slow waves offered the strongest relationship with outcome. Relatively small sample sizes may overestimate effect sizes or even produce conflicting results.

**Conclusion:**

Low power tests, generally achieved with small sample sizes, may produce misleading conclusions, especially when they are based only on *p* values and the dichotomized criteria of rejecting/not-rejecting the null hypothesis. We recommend reporting confidence intervals and effect sizes in a more complete and contextualized data analysis.

## Introduction

Traumatic brain injury (TBI) is a major cause of worldwide morbidity and mortality [1]. Identifying factors that might indicate a poorer prognosis is important for proper management of TBI patients [2]. While some predictive factors may be related to patient demographics (such as age, sex) and initial factors related to primary injury—as Glasgow Coma Scale (GCS)—other factors may be derived from physiologic variables that can reflect secondary brain injuries and, therefore, offer the possibility of informing management protocols.

The use of physiological and demographic variables as predictors of patient outcome has been largely discussed in the literature [2–5]. In particular, high time-resolution multimodal monitoring allows for an extended assessment of secondary injury after TBI [6]. However, because of difficulties in obtaining large datasets of high-resolution physiological signals, some studies have relatively small sample sizes. The failure to find a relationship between a physiological variable (or a derived index) and outcome where one truly exists (type II statistical error) could prematurely end research on a promising ‘physio-marker’. Conversely, finding a spurious relationship between a monitored variable or index when one does not exist (type I statistical error) may take significant time to be rectified in the scientific community, especially with the acknowledged ‘positive results publication bias’.

The objective of this study is to highlight potential pitfalls when only *p* values are used to interpret results from relatively small datasets. More specifically, we explored the role of sample size when physiological and demographic variables are associated with patient outcome using univariate binary logistic regression models. In this context, we also estimated the minimum sample sizes needed to obtain reproducible results.

## Methods

Data from head-injured patients having full record of monitored variables of interest, connected to a bedside computerized system (software: ICM [1992–2003], Warsaw University of Technology, Poland, and University of Cambridge, UK, and later ICM+^®^ [2003–2015] http://www.neurosurg.cam.ac.uk/icmplus Cambridge Enterprise, Cambridge, UK) with invasive monitoring of ABP and ICP over a period longer than 12 h were included in this retrospective analysis. ABP was invasively monitored through a catheter in the radial artery; the pressure transducer was zeroed at heart level. ICP was continuously monitored with Codman parenchymal probes (Johnson & Johnson Medical, Raynham, MA, USA) via a cranial access device (Technicam, Newton Abbott, UK). Probes were positioned at a constant depth in the white matter, pericontusional in focal injuries or in the nondominant frontal lobe in diffuse injuries. Patients were managed according to international TBI guidelines [7]. Patients were sedated, intubated, and ventilated. Interventions were aimed at keeping ICP < 20 mm Hg using a stepwise approach of positioning, sedation, neuromuscular paralysis, mild hyperventilation, ventriculostomy, osmotic agents, and induced hypothermia [8]. Cerebral perfusion pressure (CPP) was maintained >60 mm Hg using intravenous fluids and vasopressors. Computerized indices did not form a part of the management algorithm. The Glasgow Outcome Scale (GOS) was assessed at 6 months by outpatient assessment [9]. The digital recording of high-resolution data for further anonymous use in academic publications has been approved by the institutional ethics committee (29 REC 97/291) and local neurocritical care users’ committee.

Patients were divided between two groups, favorable (FAV) and unfavorable (UNF), according to their GOS score: 1—Death (D); 2—Persistent Vegetative State (PVS); 3—Severe disability (SD); 4—Moderate Disability (MD) and 5—Good Recovery (GR). The proportions of each GOS score for each variable are presented in Table 1.

**Table 1:** Number of patients for each variable and the proportions among different GOS scores

Variable	Outcome					Total
	*D*	PVS	SD	MD	GR	
ABP	171 (22 %)	14 (2 %)	241 (31 %)	193 (25 %)	147 (20 %)	766
Age	172 (22 %)	15 (2 %)	246 (32 %)	191 (25 %)	149 (19 %)	773
AMP	165 (22 %)	15 (2 %)	244 (32 %)	193 (25 %)	149 (19 %)	766
CPP	171 (22 %)	14 (2 %)	242 (31 %)	193 (25 %)	149 (20 %)	769
GCS	125 (22 %)	8 (1 %)	162 (29 %)	140 (25 %)	122 (23 %)	557
ICP	172 (22 %)	15 (2 %)	244 (31 %)	194 (25 %)	151 (20 %)	776
PRx	156 (22 %)	14 (2 %)	231 (32 %)	180 (25 %)	136 (19 %)	717
RAP	170 (22 %)	15 (2 %)	245 (32 %)	194 (25 %)	149 (19 %)	773
Slow	167 (22 %)	15 (2 %)	244 (32 %)	193 (25 %)	149 (19 %)	768

*D* death, *PVS* persistent vegetative state, *SD* severe disability, *MD* moderate disability, *GR* good recovery, *ABP* arterial blood pressure, *AMP* amplitude of intracranial pressure pulse, *CPP* cerebral perfusion pressure, *GCS* glasgow coma score, *ICP* intracranial pressure, *PRx* pressure reactivity index, *RAP* compensatory reserve index, *Slow* magnitude of intracranial pressure slow waves

Three different dichotomizations were used in this study: *Dicho1* contains GOS 1 for UNF and GOS 2–5 for FAV*. Dicho 2* comprises GOS 1–2 for UNF and GOS 3–5 for FAV, and finally, *Dicho 3* consist of GOS 1–3 and GOS 4–5 for UNF and FAV groups, respectively. The demographic variables used for outcome association are age GCS. The physiological variables were averaged over the entire NCCU stay. They are arterial blood pressure (ABP), intracranial pressure (ICP), amplitude of ICP pulse (AMP), magnitude of ICP slow waves (Slow), cerebral perfusion pressure (CPP), pressure reactivity index (PRx), and compensatory reserve index (RAP). There is substantial literature about these indices; for a useful description see [6].

### Bootstrapping

One of the objectives of this study is to obtain the incidence of statistically significant results when different sample sizes are used. The straightforward approach would be to consider all possible combinations of patients from the dataset, for a given sample size, and obtain the statistics for each case. However, this would be impracticable since there are approximately 10^48^ possible combinations for a sample size (*N* = 30 for example). A more appropriate approach is to use a bootstrapping method to estimate the probability distribution of the chosen statistic.

We examined samples of *N* = 20 up to *N* = 15,000. Patients for each *N* were randomly chosen with reposition. A univariate logistic regression was applied, and the odds ratio (OR) of favorable versus unfavorable outcome was obtained with its respective *p* value. This process was repeated 10,000 times for each sample size and for each variable. Thus, we can estimate the minimum sample size required to obtain a statistically significant result in 90 % of the drawings.

## Results

**Fig. 1:**
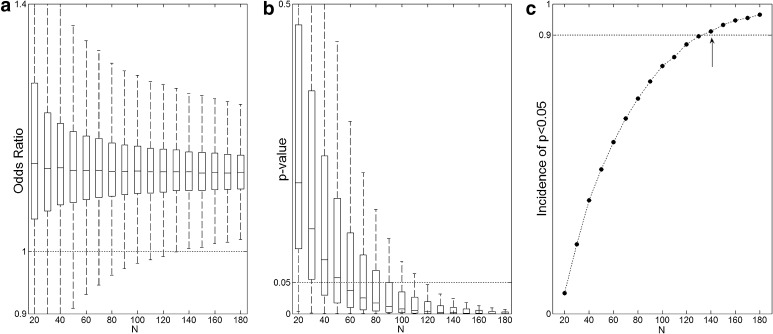
Considering ICP as the predictor variable in (**a**) *box plot* of the odds ratio and respective *p* values, in (**b**) as a function of sample size *N* obtained in 10^5^ tests, in (**c**) the incidence of *p* values below the significance level of 5 %. The larger the sample size, the better the reproducibility of the result. *Arrows* indicate the minimum sample size for ICP (*N*_min_ = 100) in order to obtain reproducible results 90 % of the time

Using ICP and dichotomization Dicho 1 as an example, Fig. 1 presents the box plots for the odds ratio (OR) as well as the *p* values for different sample sizes (*N*). For small sample sizes, the variability of OR and *p* values is larger and gets smaller with increasing *N*. The incidence of significant results increases with sample size, reaching 90 % at *N* = 140.

**Fig. 2:**
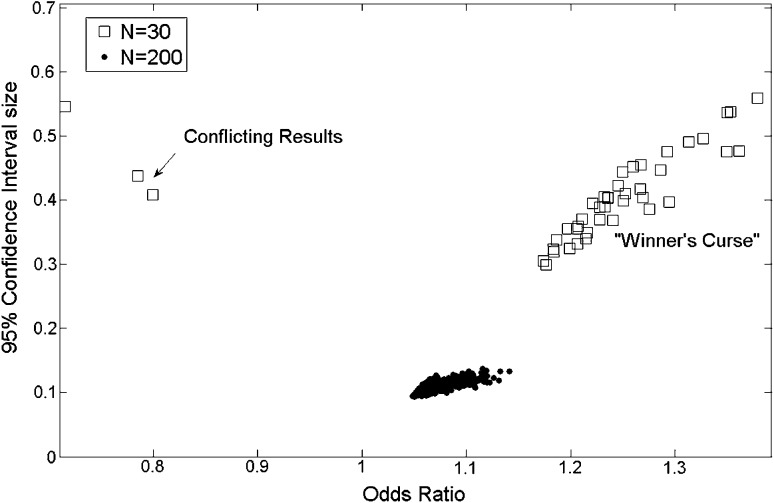
Considering only significant results (*p* < 0.05), odds ratio, and 95 % confidence interval sizes for 1000 results with sample size *N* = 30 and *N* = 200. For small sample sizes (*open squares*), the obtained effect size (OR) is overestimated and the so-called winner’s curse and the confidence intervals are larger. Also, the *arrow* points to possible conflicting results (OR < 1) that may occur when sample sizes are small

Considering only statistically significant results (*p* < 0.05), Fig. 2 compares the OR and 95 % confidence interval sizes obtained in 1000 random samples for two different sizes, *N* = 30 and *N* = 200. For *N* = 30 (open squares), the values of OR obtained are more dispersed varying from 1.2 to 1.4 and the sizes for the 95 % CI are also larger from 0.2 to 0.6. On the other hand, for *N* = 200, the values of OR varies between 1.05 and 1.15 and the 95 % CI size is around 0.1. The statistics variability for small sample sizes is responsible for producing conflicting results as pointed out by the arrow in Fig. 2. With the same sample size (*N* = 30), the odds ratio may be statistically significant below one (OR < 1) or above one (OR > 1).

The minimum sample size (*N*_min_) needed to obtain 90 % incidence of significant results for each predictor variable considered in this study is presented in Table 2. Minimum sample sizes generally decrease for more restrictive conditions for unfavorable outcome, i.e., *N*_min_ are smaller for Dicho 1 and 2. With the exception of RAP and both demographic variables (age and GCS), the addition of SD patients increases the effect sizes and therefore diminishes *N*_min_. For CPP and AMP, the inclusion of SD patients in the unfavorable group reduces considerably the associative power of those variables, with estimated *N*_min_ greater than 15,000.

**Table 2:** Estimated minimum sample sizes (*N*_min_) required for each physiological variable for 90 % incidence of *p* values below 0.05

Dicho	ABP	Age	AMP	CPP	GCS	ICP	PRx	RAP	Slow
1	1300	370	850	550	800	140	140	560	160
2	2300	380	800	450	850	155	150	660	170
3	1200	280	–	–	380	370	190	370	380

Tests were performed considering different dichotomizations between favorable and unfavorable outcomes

*Dicho 1* compares GOS 1 versus GOS 2–5, *Dicho 2* GOS 1–2 versus GOS 3–5, *Dicho 3* GOS 1–3 versus GOS 4 and 5, *ABP* arterial blood pressure, *AMP* amplitude of intracranial pressure pulse, *CPP* cerebral perfusion pressure, *GCS* glasgow coma score, *ICP* intracranial pressure, *PRx* pressure reactivity index, *RAP* compensatory reserve index, *Slow* magnitude of intracranial pressure slow waves

## Discussion

In the current analysis, PRx, ICP, and ICP slow waves offered the strongest relationship with outcome. This result highlights the importance of impaired pressure reactivity and intracranial hypertension as secondary injuries in TBI [4].

The incidence of *p* values below the significance level, obtained through bootstrapping, can be interpreted as an estimate *power* of the test, i.e., its *sensitivity*. It is well described in the literature that power increases with sample size [10]. However, studies with small sample sizes (*N* = 30–50) can be found quite frequently even in good journals and therefore low statistical power is usually employed [11]. Underpowered tests may provide a statistically significant result that not only fails when it comes to its reproducibility but also overestimates its clinical relevance; both sensitivity and positive predictive value (PPV) are low for underpowered tests [11].

For instance, in a study with *N* = 30 patients, the odds ratio that increasing ICP will increase the odds for unfavorable outcome will most likely not be statistically significant; hence, one will not have enough evidence that increased ICP is related to worse outcome. Note that this does not imply that increased ICP is not related to worse outcome; the absence of evidence is not the evidence of absence. A non-significant result just means that there is insufficient information to prove the proposition to be either true or false; more data are needed to gather more evidence against the null hypothesis. But we must keep in mind that a large enough sample will eventually produce a statistically significant result [11] and, consequently, it should be interpreted in the light of its clinical relevance. Also, a statistically significant result in this scenario will most likely overestimate its effect size and consequently its clinical relevance, the well-known “winner’s curse” [12]. In addition, the variability of OR and *p* values for small sample sizes may produce misleading conclusions. The conflicting result presented in Fig. 2, with significant result for OR > 1 and OR < 1, illustrates the point that without any additional information other than the dichotomized criteria of rejecting/not-rejecting the null hypothesis, it is difficult to come up with any meaningful conclusions and there are no means to access the reproducibility of the results.

## Limitations

The current study considered the use of a univariate logistic regression analysis for outcome association, and sample size (*N*) considered in the analysis is evenly distributed between groups. Although outcome prediction in TBI is obviously a multivariate problem, for the current analysis we wished simply to highlight the importance of considering a more complete description of the statistical results rather than just *p* values.

The particular ‘optimal sample sizes’ (*N*_min_) obtained are for illustrative purposes only, rather than a research framework, because they were constructed using data from only one research center and, therefore, may not be applicable for other datasets. Furthermore, they deal with a specific characteristic of the analysis, which is 90 % power. There are other alternative criteria to select optimal sample sizes, for instance the “planning for precision”, which calculates the sample size required for estimating the effect size to reach a defined degree of precision [13].

Rather than just prescribing a minimum sample size needed for publication of results, the current study highlights potential pitfalls when searching for physiologic indices that predict outcome [14–18]. At least in the field of TBI research, relationships between monitored variables and outcome must be carefully interpreted when sample sizes less than 100 are used. This result only reinforces the importance of multicenter studies when it comes to clinical neuroscience.

## Conclusion

Consistent with other opinion, we recommend a more complete and contextualized description of results. Sample size, effect size, power, and confidence intervals should all be considered in addition to *p* values when interpreting results from statistical inferences. Relying only on *p* values as the final word can produce misleading conclusions, especially when combined with the dichotomized criteria of rejecting/not-rejecting the null hypothesis.

## References

[1] Roozenbeek B, Maas AI, Menon DK (2013). Changing patterns in the epidemiology of traumatic brain injury. Nat Rev Neurol.

[2] Lingsma HF, Roozenbeek B, Steyerberg EW, Murray GD, Maas AIR (2010). Early prognosis in traumatic brain injury: from prophecies to predictions. Lancet Neurol.

[3] Le Roux P, Menon DK, Citerio G, Vespa P, Bader MK, Brophy GM (2014). Consensus summary statement of the international multidisciplinary consensus conference on multimodality monitoring in neurocritical care. Neurocrit Care.

[4] Le Roux P (2013). Physiological monitoring of the severe traumatic brain injury patient in the intensive care unit. Curr Neurol Neurosci Rep.

[5] Czosnyka M, Balestreri M, Steiner L, Smielewski P, Hutchinson PJ, Matta B (2005). Age, intracranial pressure, autoregulation, and outcome after brain trauma. J Neurosurg.

[6] Czosnyka M, Smielewski P, Timofeev I, Lavinio A, Guazzo E, Hutchinson P (2007). Intracranial pressure: more than a number. Neurosurg Focus.

[7] Trauma Foundation, American Association of Neurological Surgeons, Congress of Neurological Surgeons. Guidelines for the management of severe traumatic brain injury. J Neurotrauma. 2007;24(Suppl):S1–106. doi:10.1089/neu.2007.9999. 10.1089/neu.2007.9999 17511534

[8] Stocchetti N, Maas AIR (2014). Traumatic intracranial hypertension. N Engl J Med.

[9] Jennett B, Bond M (1975). Assessment of outcome after severe brain damage: a practical scale. Lancet.

[10] Cohen J, Things I (1990). Have learned (so far). Am Psychol.

[11] Button KS, Ioannidis JPA, Mokrysz C, Nosek BA, Flint J, Robinson ESJ (2013). Power failure: why small sample size undermines the reliability of neuroscience. Nat Rev Neurosci.

[12] Ioannidis JPA (2008). Why most discovered true associations are inflated. Epidemiology..

[13] Maxwell SE, Kelley K, Rausch JR (2008). Sample size planning for statistical power and accuracy in parameter estimation. Annu Rev Psychol.

[14] Nuzzo R (2014). Scientific method: statistical errors. Nature.

[15] Gardner MJ, Altman DG (1986). Confidence intervals rather than P values: estimation rather than hypothesis testing. Br Med J.

[16] Royall RM (1986). The effect of sample size on the meaning of significance tests. Am Stat.

[17] Cumming G, Fidler F, Kalinowski P, Lai J (2012). The statistical recommendations of the American Psychological Association Publication Manual: effect sizes, confidence intervals, and meta-analysis. Aust J Psychol.

[18] Halsey LG, Curran-Everett D, Vowler SL, Drummond GB (2015). The fickle P value generates irreproducible results. Nat Methods.

